# Dr. A. R. Wright

**Published:** 1900-04

**Authors:** 


### OBITUARY.

Dr. A. R. Wright, of Buffalo, one of the most prominent homeopathic physicians in this region, died in Chicago, February 24, 1900, aged 74 years. A few days before his death he went to Chicago to consult Dr. N. Senn. After reaching the city he grew worse and died within three days after leaving home. He was born in Crawford, Orange County, on October 19, 1825, received his preliminary education in the common schools and the Albany State Normal School, from which he was graduated in 1848. He taught in the Albany Academy and the public schools of Elmira until 1852, when he began the study of medicine with Dr. N. H. Warner, of Buffalo, who was one of the first homeopathic physicians to settle in this city. He entered the Buffalo Medical College, where he remained for a time, but his health failing, he set out on a voyage to India and China for its benefit. During his absence he served for two years as medical officer on one of the Peninsular and Oriental steamers, plying between Europe and Chinese ports.

With his health restored Dr. Wright attended the medical schools of Paris and came back to this country, taking his medical degree later from the Cleveland Homeopathic Medical College. He began practice in Elmira, but after a year came to Buffalo, in 1859, and had lived and practised here since that time.

In 1898 Dr. Wright was president of the American Institute of Homeopathy and he had been for years a member of the Homeopathic State Board of Medical Examiners.

Dr. Wright married, in 1864, Charlotte Crocker, of New York, who, with two daughters, the Misses Mabel and Rosalie Wright, survives him.

